# A Quantitative Method for the Study of HIV-1 and *Mycobacterium tuberculosis* Coinfection

**DOI:** 10.1093/infdis/jiac491

**Published:** 2022-12-20

**Authors:** Samantha Donnellan, Shaun H Pennington, Alessandra Ruggiero, Carmen Martinez-Rodriguez, Marion Pouget, Jordan Thomas, Steve A Ward, Georgios Pollakis, Giancarlo A Biagini, William A Paxton

**Affiliations:** Centre for Drugs and Diagnostics, Department of Tropical Disease Biology, Liverpool School of Tropical Medicine, Liverpool, United Kingdom; School of Applied Sciences, Edinburgh Napier University, Edinburgh, United Kingdom; Centre for Drugs and Diagnostics, Department of Tropical Disease Biology, Liverpool School of Tropical Medicine, Liverpool, United Kingdom; Department of Clinical Infection, Microbiology, and Immunology, Institute of Veterinary and Ecological Sciences, University of Liverpool, Liverpool, United Kingdom; Department of Neuroscience, Biomedicine, and Movement Sciences, University of Verona, Verona, Italy; Department of Clinical Infection, Microbiology, and Immunology, Institute of Veterinary and Ecological Sciences, University of Liverpool, Liverpool, United Kingdom; Department of Clinical Infection, Microbiology, and Immunology, Institute of Veterinary and Ecological Sciences, University of Liverpool, Liverpool, United Kingdom; Centre for Experimental Pathogen Host Research, University College Dublin, Dublin, Ireland; Department of Clinical Infection, Microbiology, and Immunology, Institute of Veterinary and Ecological Sciences, University of Liverpool, Liverpool, United Kingdom; Centre for Drugs and Diagnostics, Department of Tropical Disease Biology, Liverpool School of Tropical Medicine, Liverpool, United Kingdom; Department of Clinical Infection, Microbiology, and Immunology, Institute of Veterinary and Ecological Sciences, University of Liverpool, Liverpool, United Kingdom; Centre for Drugs and Diagnostics, Department of Tropical Disease Biology, Liverpool School of Tropical Medicine, Liverpool, United Kingdom; Department of Clinical Infection, Microbiology, and Immunology, Institute of Veterinary and Ecological Sciences, University of Liverpool, Liverpool, United Kingdom

**Keywords:** coinfection, drug screening, HIV-1, *Mycobacterium tuberculosis*, tuberculosis

## Abstract

*Mycobacterium tuberculosis* and human immunodeficiency virus-1 (HIV-1) syndemic interactions are a major global health concern. Despite the clinical significance of coinfection, our understanding of the cellular pathophysiology and the therapeutic pharmacodynamic impact of coinfection is limited. Here, we use single-round infectious HIV-1 pseudotyped viral particles expressing green fluorescent protein alongside *M. tuberculosis* expressing mCherry to study pathogenesis and treatment. We report that HIV-1 infection inhibited intracellular replication of *M. tuberculosis* and demonstrate the therapeutic activity of antiviral treatment (efavirenz) and antimicrobial treatment (rifampicin). The described method could be applied for detailed mechanistic studies to inform the development of novel treatment strategies.

*Mycobacterium tuberculosis* is an obligate, acid-fast, intracellular bacillus, causing the human disease tuberculosis. Human immunodeficiency virus (HIV-1; HIV) is a lentivirus that causes acquired immune deficiency syndrome (AIDS) in humans. Both diseases are among the leading causes of death worldwide [1].

People with HIV are up to 27-times more likely to develop active tuberculosis than people without HIV and, in 2020, of the 1.3 million deaths attributed to tuberculosis, 214 000 were among people with HIV[2]. Infection with either pathogen can reactivate latent infection of the other, and antimicrobials targeting *M. tuberculosis* are known to interact with antivirals targeting HIV [3].

Despite the clinical significance of HIV and *M. tuberculosis* coinfection, our understanding of the cellular pathophysiology and therapeutic pharmacodynamics is limited. This is in part due to the absence of quantitative methods required for study of coinfection. The development of *M. tuberculosis* and HIV that carry fluorescent molecules, potentially allows for the simultaneous quantification of both pathogens using fluorescence-based modalities suitable for single-cell study and drug discovery [4, 5].

We have previously used single-round infectious pseudotyped viral particles (PVPs) to monitor antibody neutralization of Ebola virus and for the study the glycolipid composition of pathogenic and nonpathogenic *M. tuberculosis* [6, 7]. Here, we have applied a similar approach to study *M. tuberculosis* and HIV coinfection in THP-1 monocyte-derived macrophages. We utilized fluorometry, high-content imaging, and flow cytometry to assess the impact of antiviral and antimicrobial treatment on HIV and *M. tuberculosis* coinfection.

## METHODS

### Cell Culture

THP-1 cells were cultured in T75 flasks in Roswell Park Memorial Institute (RPMI)-1640 supplemented with L-glutamine, NaHCO_3_, 10% heat-inactivated fetal bovine serum (HI-FBS), and 100 U/mL penicillin/streptomycin, at 37°C with 5% CO_2_. For differentiation, THP-1 cells were seeded in 96-well plates (PerkinElmer) at 1 × 10^6^ cells/well (200 µL/well) in FluoroBrite Dulbecco’s Modified Eagle’s Medium (DMEM) supplemented with 10% HI-FBS, L-glutamine, and 100 ng/mL phorbol 12-myristate 13-acetate (PMA; Sigma) at 37°C with 5% CO_2_.

HEK-293 T cells (American Type Culture Collection [ATCC]: CRL1573) and TZMbl cells (National Institute for Biological Standards and Control [NIBSC]: ARP5011) were cultured in DMEM supplemented with 10% FBS and 100 U/mL penicillin/streptomycin, at 37°C with 5% CO_2_.

### HIV-1-PVP Production

HIV-1-PVPs were produced by transfection of HEK-293T cells. One backbone plasmid expressing HIV-1 structural and polymerase proteins (pNL4-3; NIBSC: ARP12455; 2 µg/10-cm dish) along with a plasmid expressing either the HIV-1-BAL envelope protein (NIBSC: 140244; 2 µg/10-cm dish) or the vesicular stomatitis virus glycoprotein (VSV-G) envelope protein (NIBSC: 4693; 2 µg/10-cm dish). At 48 hours posttransfection, supernatant containing virus was collected, stored at −80°C and used for downstream applications. HIV-1-PVP (HIV-PVP) titers were quantified by measuring HIV-p24 protein concentration (Aalto) and virus infectivity on TZMbl cells prior to use.

### *M. tuberculosis* Culture

H37Rv-mCherry contains an integrative plasmid (pvv16) carrying Rv2170 constitutively expressed from a HSP60 promoter (kind gift from Professor David Russell, Cornell University). Growth curves were generated according to standard procedures. Aliquots of *M. tuberculosis* H37Rv-mCherry were cultured aerobically at 37°C in 7H9 liquid media (7H9 broth, supplemented with 0.05% [v/v] Tween-80, 0.2% [v/v] glycerol, 10% oleic acid-albumin-dextrose-catalase, and 50 µg/mL hygromycin). The optical density of cultures was measured at 600 nm every 24 hours for 7 days. In parallel, colony forming units were enumerated by serial dilution and inoculation on solid media (Middlebrook 7H11 agar, supplemented with 10% oleic acid-albumin-dextrose-catalase, 0.2% [v/v] glycerol and 0.05% [v/v] Tween-80). Cultures were maintained in mid-log phase, and concentrations adjusted for infection based on growth curves.

### HIV and *M. tuberculosis* Coinfection and Treatment

PVPs were added to macrophages at a concentration 30 ng/well in FluoroBrite DMEM, supplemented with 10% HI-FBS and L-glutamine (200 µL/well), as appropriate. PVPs lacking the envelope protein (ΔEnv), making them unable to enter target cells, were included at 30 ng/well as a negative control. Concentrations were selected based on our previously published work [7]. After 24 hours, cell media were removed, and the macrophages infected with *M. tuberculosis* at a multiplicity of infection (MOI) of 1:5. The MOI was selected based on our previously published work [8]. After 24 hours, the cells were washed to remove extracellular baccili. Efavirenz in FluoroBrite DMEM at 10 µg/mL was added to THP-1 cells 2 hours prior to infection with HIV and remained in the culture media for the duration of the experiment, as appropriate. Rifampicin in FluoroBrite DMEM at 10 µg/mL was added 24 hours after the addition of *M. tuberculosis*, as appropriate.

### Confocal Laser Scanning Microscopy Live Imaging

Plates containing infected cells were sealed with Breath-EASIER sealing membranes (Sigma) and wrapped in parafilm. Plates were then wiped with 5% surfanios, then 70% ethanol and transferred to the confocal microscope (LSM 880; Zeiss). Z-stacks were obtained at 60× magnification.

### Fluorometry

Infected plates were sealed with Breath-EASIER sealing membranes (Sigma) and wrapped in parafilm. Plates were then wiped with 5% surfanios, then 70% ethanol and transferred to the plate reader (Varioskan LUX). Fluorescence was measured at 395/409 nm (excitation/emission) for green fluorescent protein and 587/610 nm for mCherry.

### Flow Cytometry

THP-1 cells were washed twice with prewarmed phosphate-buffered saline (PBS) and then detached from the plate using ice-cold PBS and transferred to screw caped tubes. Cells were stained for viability (Pacific Blue; LifeTechnologies), washed, and then incubated in 5% paraformaldehyde at room temperature for 2 hours. Cells were washed once, resuspended in ice-cold PBS, and stored in the absence of light until acquisition using a FACS LSR II flow cytometer (BD Biosciences). Standard procedures were used to maintain the instrument and quality control performed daily. A compensation matrix was created, and sequential cell isolation used to identify populations using FlowJo version 10 (Treestar Inc).

## RESULTS

### Coinfection of THP-1 Macrophages by HIV and *M. tuberculosis*

**Figure 1.**
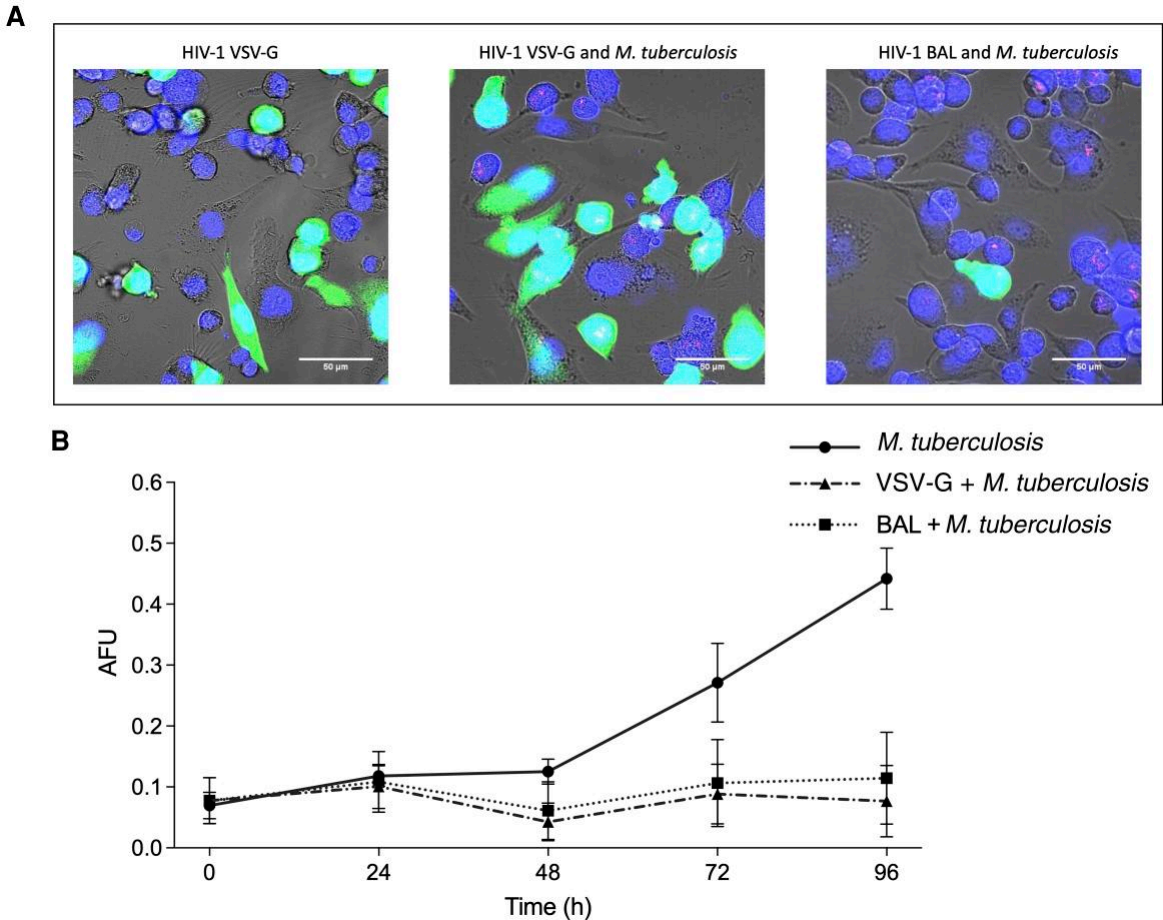
The impact of HIV-1 (HIV) infection on the growth of intracellular *Mycobacterium tuberculosis* over 96 hours in THP-1 macrophages. *A*, Live-cell confocal imaging of HIV and *M. tuberculosis* coinfection in THP-1 macrophage cultures singularly infected with HIV-VSV-G, infected simultaneously with HIV-VSV-G and *M. tuberculosis*, and infected simultaneously with HIV-BAL and *M. tuberculosis*. The green signal (green fluorescent protein) indicates host-cell infection by HIV. The red signal (mCherry) indicates host-cell infection by *M. tuberculosis*. All images were acquired 72 hours postinfection at 63× magnification. Scale bars represent 50 µm. Images are representative of 3 individual experiments. *B*, The impact of HIV infection on the growth of intracellular *M. tuberculosis* over 96 hours in THP-1 macrophages based on the detection of mCherry (587/610 nm [excitation/emission]) in AFU. Data are shown for THP-1 macrophage cultures infected with *M. tuberculosis* alone (closed circles; solid line), HIV-VSV-G and *M. tuberculosis* (closed triangle; dashed and dotted line) or HIV-BAL and *M. tuberculosis* (closed square; dotted line); n = 3. Error bars represent the standard deviation of the mean. Abbreviations: AFU, arbitrary fluorescent unit; HIV-1, human immunodeficiency virus-1; VSV-G, vesicular stomatitis virus glycoprotein.

We first confirmed by confocal microscopy that intracellular coinfection had been established (Figure 1*A*). For cultures infected with HIV-VSV-G-PVP and *M. tuberculosis,* as well as for cultures infected with HIV-BAL-PVP and *M. tuberculosis*, coinfection was observed amongst THP-1 macrophages (Figure 1*A*). Visual inspection revealed that HIV-VSV-G-PVP had a higher infectivity rate than the HIV-BAL-PVP (Figure 1*A*).

Fluorometry was utilized to study the impact of HIV infection on *M. tuberculosis* growth kinetics. Over the course of 96 hours, in the absence of HIV-PVP, intracellular replication of *M. tuberculosi*s was observed (Figure 1*B*). In cultures where HIV-VSV-G or HIV-BAL-PVP infection had previously been established, there was no detectable growth of *M. tuberculosi*s (Figure 1*B*).

### Single-Cell Assessment of *M. tuberculosis* Growth Kinetics in Macrophages Infected With HIV

**Figure 2.**
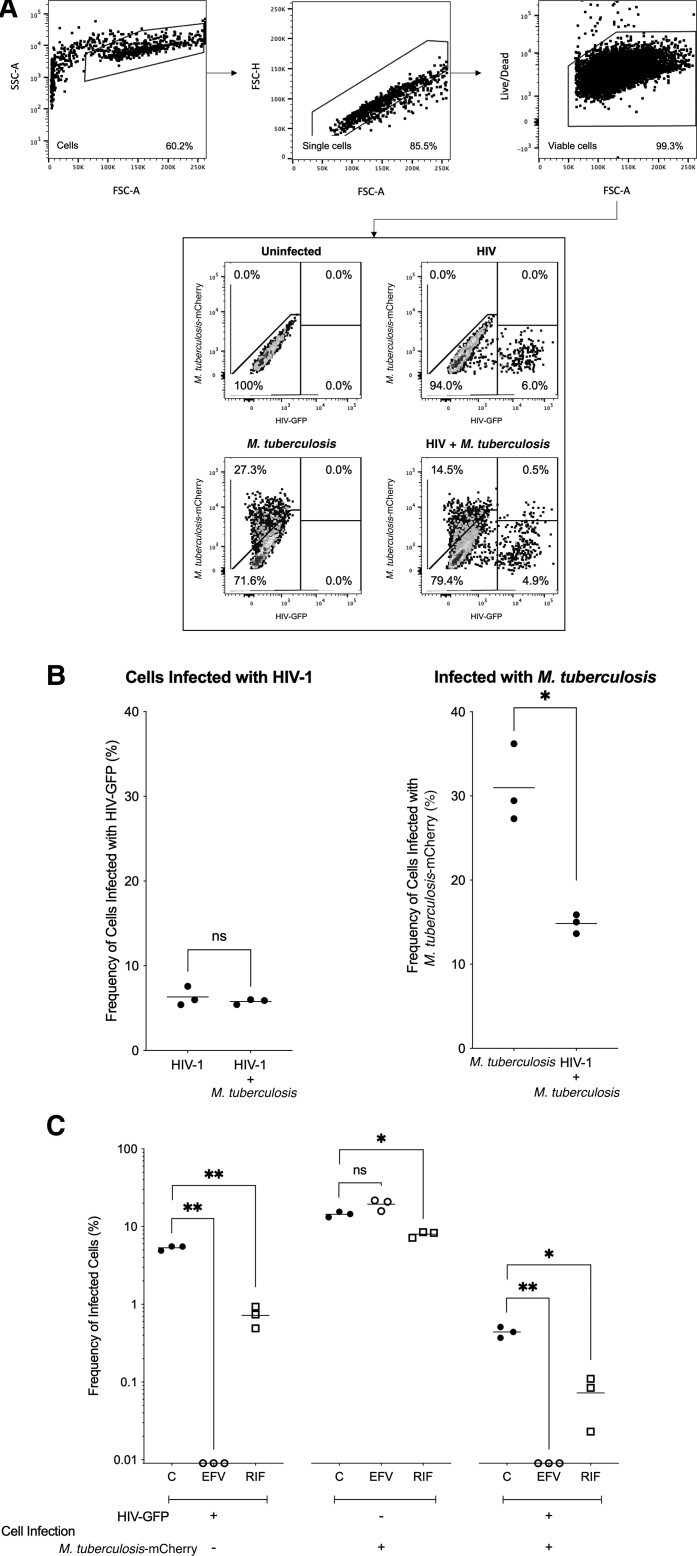
The impact of coinfection on the relative frequency of HIV-1 (HIV) and/or *Mycobacterium tuberculosis* infection in THP-1 cells. *A*, Cells were identified based on their size and granularity and single cells identified based on the proportionality of area and height. Dead cells were removed by staining for viability (Live/Dead) and gating on the negative population. mCherry was used to identify *M. tuberculosis*-infected cells and GFP was used to identify HIV–infected cells. Representative dot plots are shown for THP-1 macrophages recovered from uninfected cultures, macrophages cultured with HIV-VSV-G, macrophages cultured with *M. tuberculosis*, and macrophages cultured with both HIV and *M. tuberculosis*. *B*, Frequency of cells infected with HIV or *M. tuberculosis* for THP-1 macrophage cultures singularly infected with HIV or *M. tuberculosis*, or for cultures infected simultaneously with HIV-VSV-G and *M. tuberculosis* is shown; n = 3. *C*, For untreated control cultures and cultures treated with efavirenz or rifampicin, the frequency of cells singularly infected with HIV, singularly infected with *M. tuberculosis,* or simultaneously infected with HIV and *M. tuberculosis* is shown; n = 3. Statistical comparisons were made using unpaired *t* tests. * *P* < .05, ** *P* < .005. Abbreviations: C, control; EFV, efavirenz; FSC, forward scatter; GFP, green fluorescent protein; HIV-1, human immunodeficiency virus-1; ns, not significant; RIF, rifampicin; SSC, side scatter; VSV-G, vesicular stomatitis virus glycoprotein.

To improve assay resolution, we performed single-cell analysis by flow cytometry. A sequential gating strategy was used to identify THP-1 cells infected with virus and/or bacilli (Figure 2*A*). In cultures singularly infected with *M. tuberculosis*, approximately 31% of viable cells were infected with *M. tuberculosis* and, in cultures where HIV infection had been established prior to the addition of *M. tuberculosis,* approximately 15% of viable cells were infected with *M. tuberculosis* (*P* = .0378; Figure 2*B*). The addition of *M. tuberculosis* had no impact on the frequency of HIV infection (Figure 2*B*).

### Single-Cell Assessment of Drug Activity in Coinfection

We next set out to demonstrate the utility of this method for the assessment of antiviral and/or antimicrobial treatment. Incubation with efavirenz was shown to block HIV-PVP infection (Figure 2*C*); approximately 5% of viable cells were singularly infected with HIV in untreated control cultures and no cells were infected in cultures treated with efavirenz (*P* = .0015); approximately 0.4% of viable cells were simultaneously infected with HIV and *M. tuberculosis* in untreated control cultures and no cells were identified in cultures treated with efavirenz (*P* = .0083; Figure 2*C*). Efavirenz had no impact on the frequency of cells singularly infected with *M. tuberculosis* (Figure 2*C*).

Incubation with rifampicin was shown to have a significant impact on the intracellular burden of *M. tuberculosis* (Figure 2*C*); approximately 14% of viable cells were singularly infected with *M. tuberculosis* in untreated control cultures and approximately 8% were infected in cultures treated with rifampicin (*P* = .0236); approximately 0.4% of viable cells were simultaneously infected with HIV and *M. tuberculosis* in untreated control cultures and approximately 0.07% were identified in cultures treated with rifampicin (*P* = .0123; Figure 2*C*). Rifampicin was also shown to reduce the frequency of cells singularly infected with HIV; approximately 5% of viable cells were infected in untreated control cultures and approximately 0.7% of viable cells were infected in cultures treated with rifampicin (*P* = .0031; Figure 2*C*).

## DISCUSSION

Here, we describe a quantitative platform suitable for the study HIV and *M. tuberc*ulosis coinfection. The described method may be applied to study disease pathogenesis and could serve to inform the development of novel treatment strategies targeting either pathogen alone, or both pathogens simultaneously.

Interestingly, and consistent with published literature [9], prior exposure to one pathogen was observed to influence the response to another subsequently encountered pathogen. Specifically, infection of macrophages with either HIV-VSV-G-PVP or HIV-BAL-PVP, prior to *M. tuberculosis* infection, resulted in sustained inhibition of intracellular growth of *M. tuberculosis*.

Pathogens can modulate cellular metabolism through direct interaction with Toll-like receptors via pathogen-associated molecular patterns or through indirect activation of innate immune signaling pathways. Because it is well-established that HIV infection drives M1 polarization in vitro [10], it is reasonable to hypothesize that proinflammatory polarization may have impacted the capacity for *M. tuberculosis* to replicate intracellularly. Interestingly, because this effect was observed for both HIV-VSV-G-PVP and HIV-BAL-PVP, it can be assumed that the mechanism is independent of the viral envelope. Given that *M. tuberculosis* is also known to differently influence macrophage polarization [11], the order in which cells encounter either HIV or *M. tuberculosis* could very well influence the dynamics of disease progression and further study is warranted.

It would be an oversimplification to attempt to infer any link between the observations made here and clinical disease progression. The model presented here is unlikely to provide insight into long-term disease progression—it does, however, represent a unique platform to study the earliest stages of coinfection where HIV is already established, and where *M. tuberculosis* is subsequently encountered. An appreciation of recent evidence concerning macrophage heterogeneity, in respect to both development and metabolism, indicates that the true pathophysiological representation of polarization is far more complex [12]. Whilst simplistic definitions of M1 and M2 macrophage polarization are useful, data presented elsewhere have demonstrated that, in reality, multiple distinct macrophage activation states exist across as spectrum [12].

Recent evidence suggests that the developmental origin of macrophages can determine their responses to infection stresses. In support, metabolic and transcriptional studies have demonstrated that embryonically derived alveolar macrophages and hematopoietically derived interstitial macrophages respond differently to *M. tuberculosis* [13]. Despite this, the significance of the observations made here should not be overlooked—reduced intracellular replication of *M. tuberculosis* is likely to prolong the time taken to sterilize the intracellular space through antimicrobial treatment and may increase risk of treatment failure.

We have demonstrated the potential utility of this platform for assessing the activity of antivirals and antimicrobials targeting HIV and *M. tuberculosis*. Efavirenz is known to block the integration of viral genomic RNA and was shown to completely block HIV infection. Similarly, rifampicin resulted in a reduction in the frequency of macrophages infected with *M. tuberculosis*. Interestingly, the addition of rifampicin was also shown to impact the frequency of cells identified as being singularly infected with HIV. Rifampicin is known to inhibit the DNA-dependent RNA polymerase of bacteria and viruses and has previously been shown to inhibit viral assembly of DNA viruses and the reverse transcriptase, RNA-directed DNA polymerase, of the Rous sarcoma virus [14, 15].

It was striking that off-target effects were observed following treatment with single compounds. Given that tuberculosis and HIV treatment requires administration of multiple compounds simultaneously, we would encourage further work to understand the cellular pathophysiological impact of typical combination therapeutic strategies in the context of coinfection. Our platform affords an opportunity to assess novel combination strategies that seek to investigate potential synergistic interactions between antiviral and antimicrobial as well as host-directed immunomodulatory therapeutics. Further value could be added through inclusion of concentration-responses analysis and/or through use of primary host cells, including monocyte-derived macrophages and primary macrophages isolated from healthy volunteers and people with HIV. It is possible that macrophages with distinct developmental origins could show distinct phenotypes with respect to *M. tuberculosis* infectivity and intracellular growth.

## Notes

***Financial support.*** This work was supported by the Medical Research Council (grant numbers G1002586, MR/N028376/1, and MC_PC_14111 to G. A. B. and S. A. W.); the Wellcome Trust (grant number 105620/Z/14/Z to G. A. B. and S. A. W.); UK Research and Innovation Strength in Places Fund (grant number SIPF 20197 to G. A. B. and S. A. W.); and the European Community Seventh Framework Programme (grant number HEALTH-F3-2012-305578 to G. P. and W. A. P.). Funding to pay the Open Access publication charges for this article was provided by UK Research and Innovation Strength in Places Fund and the European Community Seventh Framework Programme.
